# Rhupus arthropathy as the presenting manifestation in Juvenile SLE: a case report

**DOI:** 10.1186/1546-0096-5-7

**Published:** 2007-05-04

**Authors:** Erbil Unsal, Ayse Ozgun Arlı, Hakkı Akman

**Affiliations:** 1 Dokuz Eylul University, Faculty of Medicine, Department of Pediatrics, Division of Immunology-Rheumatology, Balcova 35340 Izmir, Turkiye

## Abstract

An 8.5-year-old girl was referred with swelling of both knees lasting for two years. ANA was found as negative. She was diagnosed as oligoarticular JIA. After two years of follow-up, thrombocytopenia was detected during routine screening. Her ANA and anti ds-DNA antibodies also became positive, with low levels of C3 and C4. She was diagnosed as Juvenile SLE, meeting the criteria cytopenia, positive immunoserology (anti dsDNA), positive ANA test, and four years of ongoing chronic arthritis, so called as "rhupus arthropathy". We should be aware of the several initial incomplete presentations of lupus in children. We should be careful in monitoring the serious manifestations of the disease in juvenile lupus patients with rhupus arthropathy, and consider the poor response to standard disease modifying agents.

## Background

Systemic lupus erythematosus (SLE) is an episodic, multisystem, autoimmune rheumatic disease characterized by diversity of both clinical and immunological abnormalities [1,2]. Arthralgia and arthritis affect the majority of children with SLE [1]. The arthritis is characteristically short in duration, lasting 24 to 48 hours, and can be migratory [1]. In some children, the arthritis is persistent and is characterized by swelling, tenderness, and loss of range of motion. Although the synovitis of SLE may be minimally proliferative, it is only occasionally erosive and usually does not result in permanent deformity [1]. SLE can mimic JIA, especially when it is presented as chronic and erosive arthritis. A young lady with chronic oligoarthritis is presented here, whose clinical picture eventually turned out to be typical lupus.

## Case report

**Figure 1:**
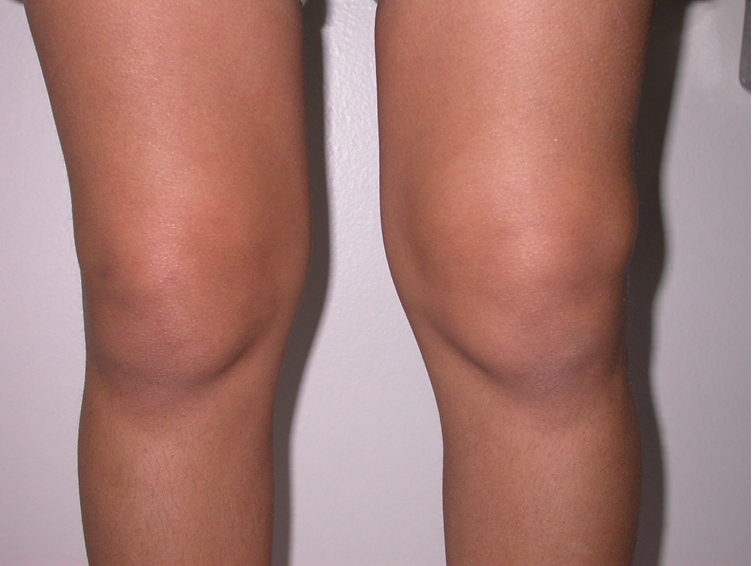
Juvenile SLE with buggy synovitis.

**Figure 2:**
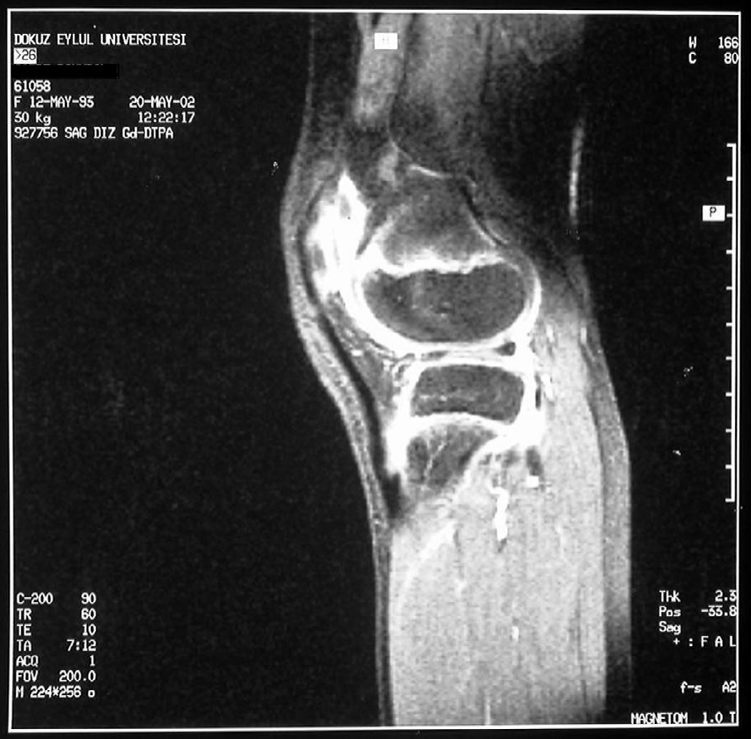
Contrast enhanced MRI of the right knee demonstrating chronic inflammation with synovial thickening.

**Figure 3:**
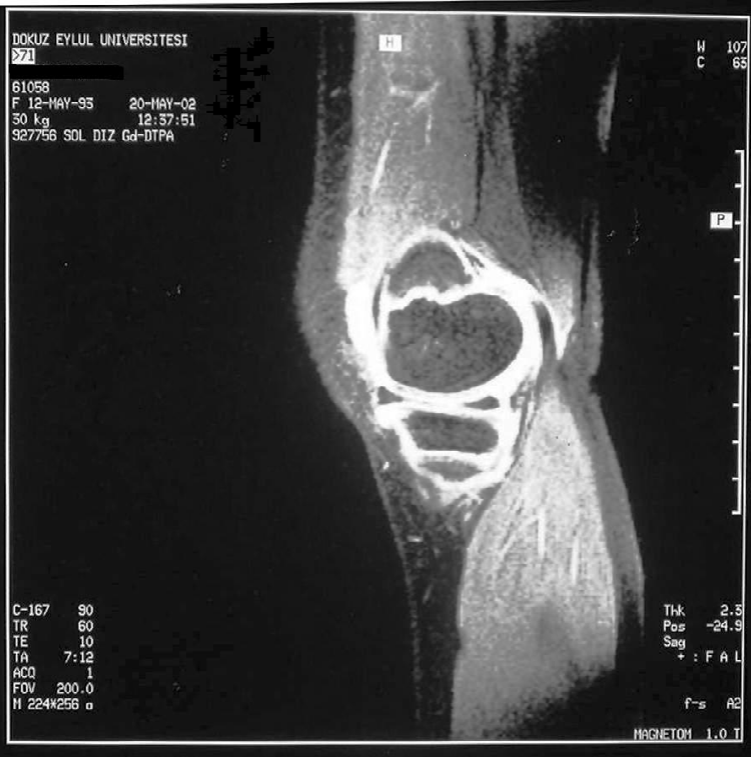
Contrast enhanced MRI of the left knee demonstrating chronic inflammation with synovial thickening.

An 8.5 year-old-girl was referred with swelling of both knees lasting for two years. She did not have complaints in any other joints. On her first examination, she was growing well. She had bilateral effusions of the knees (figure 1), and the other joints and systems were normal. MRI of both knees with gadolinium demonstrated chronic inflammation with synovial thickening (figure 2 and 3), suggesting erosive arthritis. Initial laboratory results revealed normal complete blood count (CBC), urine analysis, complement levels and immunoglobulin levels. ANA was also negative. She was diagnosed as oligoarticular JIA; naproxen was commenced, and she was regularly followed at 3 month intervals. She did not have any symptoms consistent with lupus such as alopecia, facial rash or oral ulcers. After two years of follow-up, she had a flare-up of arthritis in the knees, and epistaxis at the age of 10.5 years. Laboratory results revealed thrombocytopenia (23,000/mm^3^), coombs negative anemia (Hb: 10.7 g/dl), and normal WBC count (5900/mm^3^). ANA was positive (1/1280, speckled pattern), anti-dsDNA level was 1/20 positive, rheumatoid factor level was 9.88 IU/ml (0–14 IU/ml), C3 (75.7 mg/dl) and C4 (5.4 mg/dl) levels were low. Antiphospholipid antibodies were negative. Tests for anti-extractable nuclear antigens (ENA) (SSA, SSB, SM, SM/RNP, SCL-70 and Jo-1), were also negative. She did not have proteinuria. The antibodies for Ebstein Barr virus, parvo virus B19, and rubella were negative. She also had a palpable thyroid gland. Thyroid function tests were suggestive of Hashimoto thyroiditis: Free T3: 3.87 pg/ml (1.8–4.2 pg/ml), free T4: 1.36 ng/dl (0.8–1.9 ng/dl), TSH: 12 uIU/ml (0.4–5.0 uIU/ml), ATG > 3000 IU/ml (0–50 IU/ml), ATA: 455 IU/ml (0–50 IU/ml). She was diagnosed as Juvenile SLE, after four years of ongoing chronic arthritis. Prednisone (1 mg/kg/day) and methotrexate (MTX)(15 mg/m^2^/week) were started. She was under control within the first month, complement levels became normal and anti-dsDNA became negative. Prednisone was gradually reduced to low dose. She had a systemic flare of lupus at the age of 12, with high fever, alopecia, arthritis of both wrists and left third PIP. Direct Coombs IgG became positive without overt hemolytic anemia. She was under control with oral prednisone (1 mg/kg/day). She is now thirteen years old. A recent exacerbation of arthritis in the knees with no other joint involvement was controlled with intra-articular steroids. RF is still negative. ENA tests are also negative. She is on MTX treatment (15 mg/week) with oral prednisone (5 mg/day).

## Discussion

Systemic lupus erythematosus ranges from an insidious, chronic illness with history of intermittent signs and symptoms to an acute and rapidly fatal disease [1]. Articular symptoms are the most common clinical manifestation of SLE [3,4]. Joint involvement occurs in approximately 90% of patients at sometime during the course of their disease [3,5]. Arthritis commonly involves the small joints of the hands, wrists, elbows, shoulders, knees, and ankles [1]. The degree of involvement may range from minor arthralgia to severe deforming arthritis [4]. Joint pain is often severe even though objective findings are minimal [1]. Although joint pain and swelling are common features of SLE, erosive arthritis is generally reported rarely (<5%) in patients [2].

In the historical evaluation of SLE patients with chronic arthritis, Fallet mentioned a familial coexistence of chronic polyarthritis and lupus in 1963 [6]. Kantor reported a case in 1969 having both RA (Rheumatoid Arthritis) and SLE [7]. Labowitz et al [3] reviewed 25 SLE patients retrospectively in 1971, and six of them were found to have persistent arthritis lasting from six to twelve months, and they were first diagnosed as RA. Brzezinska reported a case with coexistence of RA and SLE in 1973 [8], and Meijers [9] presented a case with chronic synovitis as an unusual manifestation of SLE, in 1978. Fishman [10] in 1981 and Isenberg [11] in 1982 reported cases, which had RA as an initial diagnosis, and they were classified as SLE after many years. Tauchi demonstrated the coexistence of the two diseases in 2 sisters, who also had Marfan syndrome, in 1985 [12]. Venegoni reported a 29-year-old white female in 1987, who developed SLE with diffuse proliferative glomerulonephritis following long standing RA [13]. Cohen et al [14] reported 11 patients with RA and SLE in 1987, which showed that the coexistence of two diseases was not rare as it was thought to be. They suggested that erosive arthritis was not a feature of SLE, but concomitant RA. In the same year, Martini [15] reported an 11-year-old girl in 1987, who was initially diagnosed as JRA at the age of five. She developed SLE after 6 years, and the author pointed out that Jaccoud's type chronic arthropathy might be an initial manifestation of SLE. Panush et al [16] reviewed children who had both SLE and JRA in 1988. They were adolescent girls with polyarthritis, subcutaneous nodules, malar or discoid type rashes, photosensitivity and nephritis. They were found to have erosive arthritis and RF positivity, along with ANA positivity and hypocomplementemia. They had autoimmune type thrombocytopenia. Our patient also had the same type of thrombocytopenia; however, she did not have RF positivity. It might be due to her younger age at onset. Cassidy stated that RF was found as positive in children with later age of disease onset and polyarticular disease. They might be a result rather than a determining event in children who will have disabling disease course in their early adulthood years [17]. In a study by Segovia et al [18] in 1988, 858 patients with SLE were reviewed, and 41 of them were found to have deformities of the hands. These patients had more rheumatoid factor (RF) positivity with sicca symptoms, and anti ds-DNA antibodies. Brand [19] reported 11 patients in 1992 that fulfilled the criteria of both RA and SLE. All patients had a symmetrical small joint polyarthritis and features of SLE such as rash, photosensitivity, oral ulceration, serositis, cytopenia, and biopsy proven lupus nephritis. Eight had hypocomplementemia. Autoantibodies were characteristic of the two diseases: all patients had rheumatoid factor and antibodies to double stranded DNA, eight (73%) had antibodies to collagen, and five (46%) had antibodies to Ro (SS-A). There was also an overlap of HLA phenotypes. Six patients were DR4 and seven were DR2 or DR3 positive, and of the five patients who were DR4 negative, four shared class I alleles often associated with DR4. He suggested if the cases had both RA and SLE, they shared a common autoimmune dysfunction. Takei et al reported two cases with SLE and MCTD in 1997, who were first diagnosed as JRA [20], and after 8 years their ANA and anti ds-DNA levels became positive with the onset of clinical picture of SLE, a similar picture as our patient. Eisenberg et al [21] documented the long term follow-up of 21 patients with systemic onset JIA in 1999, five of them developed chronic destructive arthritis, and one of those had SLE after eight years.

Panush first used the term 'rhupus syndrome' in 1988 to describe patients with overlapping signs and symptoms of RA and SLE. He mentioned that it was unclear whether patients with rhupus represented a distinct clinical and immunologic entity, or the coincidental occurrence of RA and SLE [16]. Simon et al [22] in 2002 described 22 Mexican patients with rhupus, all of them had erosive arthritis; they also demonstrated that HLA-DR1 and HLA-DR2 alleles were significantly increased in this group. Ostendorf [23] in 2003 demonstrated the difference between Rhupus and Jaccoud's arthropathy, by demonstrating the destructive character of Rhupus arthropathy in MRI. Fernandez et al [24] in 2004 reviewed the 'historical evolution' of lupus arthropathy. They described rhupus arthropathy as one of the serious articular involvement of lupus, rather than a superposition, or overlap of RA and SLE. Recently, they published a series of 8 patients with rhupus arthropathy between 17 and 38 years at the disease onset [25] in 2006. Seven of them were women, and the diagnosis was RA in 7 patients (including the male patient) at the beginning. They developed SLE after an average of 2.8 years (range: few yrs. to 5 years). Similarly, SLE was diagnosed after four years of rhupus arthropathy in our patient. The therapeutic response to disease modifying agents was not as successful as the ones in classical RA. More important, serious manifestations of SLE were common, such as glomerulonephritis and CNS disease with seizures. The importance of serum anti-cyclic citrullinated peptide antibodies (anti-CCP) levels in patients with RF positivity and/or in patients with erosive joint disease was highlighted by Low [26], Kasapcopur [27] and Kwok [28]. They found high levels of anti-CCP antibodies in polyarticular JIA patients with erosive arthritis. Recently, Amezcua-Guerra [29] studied the prevalence of antibodies against anti-CCP antibodies in rhupus. They categorized rhupus as an overlap between RA and SLE, because of the positivity of anti-CCP, anti-dsDNA and anti-Sm antibodies in the related patients.

In conclusion, our patient has the rare features of SLE as rhupus arthropathy. We should be careful in monitoring the serious manifestations of the disease in juvenile lupus patients with rhupus arthropathy, and consider the poor response to standard disease modifying agents.
